# Coal Gasification Slag-Derived Ceramsite for High-Efficiency Phosphorus Removal from Wastewater

**DOI:** 10.3390/nano15231822

**Published:** 2025-12-01

**Authors:** Yu Li, Ruifeng Wang, Kexuan Shen, Yi Ye, Hui Liu, Zhanfeng Yang, Shengli An

**Affiliations:** 1 Key Laboratory of Green Extraction and Efficient Utilization of Light Rare-Earth Resources, Inner Mongolia University of Science and Technology, Ministry of Education, Baotou 014010, China; yul017@oit.edu.cn (Y.L.); shenkx123456@163.com (K.S.); yy840652704@163.com (Y.Y.); yuxingliuhui@163.com (H.L.); yang_zhanfeng@163.com (Z.Y.); 2 Inner Mongolia Key Laboratory of Advanced Ceramic Materials and Devices, School of Materials Science and Engineering, Inner Mongolia University of Science and Technology, Baotou 014010, China; 3 School of Chemical Engineering, Ordos Institute of Technology, Ordos 017010, China; rfwang@oit.edu.cn; 4 Inner Mongolia Environmental Governance Engineering Co., Ltd., Hohhot 01000, China; 5 State Key Laboratory of Balyunobo Rare Earth Resources and Comprehensive Utilization, Baotou 014010, China

**Keywords:** coal gasification slag, phosphorus removal, sintered ceramsite, gehlenite

## Abstract

Coal gasification slag (CGS), an industrial solid waste produced during high-temperature (1200–1600 °C) coal gasification, was utilized as the primary raw material, combined with minor additions of coal gangue and calcium oxide, to synthesize ceramsite filter via high-temperature sintering (900–1160 °C) for phosphorus-containing wastewater treatment. The resulting ceramsite was evaluated for compressive strength, apparent porosity, water absorption, mineral phase composition, hydrolysis properties, and phosphorus removal performance. Experimental results revealed that increasing sintering temperature and calcium oxide content shifted the dominant crystalline phases from anorthite and hematite to gehlenite, anorthite, wollastonite, and esseneite, promoting the formation of porous structures. This transition increased apparent porosity while reducing compressive strength. Under optimal conditions (1130 °C, 20 wt.% CaO, 1 h sintering), the ceramsite (CM-20-1130) exhibited an apparent porosity of 43.12%, compressive strength of 3.88 MPa, apparent density of 1.084 g/cm^3^, and water absorption of 33.20%. The high porosity and abundant gehlenite and wollastonite phases endowed CM-20-1130 with enhanced hydrolysis capacity. Static phosphorus removal experiments demonstrated a maximum phosphorus removal capacity of 2.77 mg/g, driven by the release of calcium and hydroxide ions from gehlenite and wollastonite, which form calcium-phosphate precipitates on the ceramsite surface, enabling efficient phosphorus removal from simulated wastewater.

## 1. Introduction

Phosphorus pollution poses a significant threat to global freshwater systems, contributing to eutrophication, a process that accelerates organic matter accumulation, triggers algal blooms, and promotes bacterial proliferation, ultimately compromising ecological integrity and water quality in the world [1,2,3,4,5,6]. Sources of phosphorus pollution include agricultural runoff, municipal wastewater, and industrial effluents from sectors such as steel, chemical, pharmaceutical, paper, dyeing, food processing, semiconductor, and lithium battery manufacturing. Eutrophication exacerbates water resource scarcity, impacting human health, economic stability, and societal development [7,8]. To mitigate this issue, stringent regulations have been established globally. For instance, Dutch guidelines limit phosphate concentrations in discharged effluents to 0.15 mg/L (calculated in terms of TP), while the U.S. Environmental Protection Agency (EPA) set a threshold of 0.1 mg/L (calculated in terms of TP) for reservoirs and lakes [9,10]. Australia sets an even stricter limit for phosphorus concentration entering such waters, requiring it to not exceed 0.026 mg/L (calculated in terms of TP) [11], and China’s Integrated Wastewater Discharge Standard (GB8978-1996) specifies phosphate limits of ≤0.5 mg/L (calculated in terms of TP). Therefore, effective phosphorus removal from wastewater is critical to meeting these standards and protecting aquatic ecosystems. Current phosphorus removal technologies include chemical precipitation, crystallization, adsorption, membrane separation, electrochemical, and biological approaches [6,12,13,14,15,16,17,18]. Among these, adsorption stands out as a prominent approach in wastewater treatment due to its compact infrastructure requirements, exceptional phosphorus removal efficiency, and simple process [19]. The key to phosphorus removal by adsorption lies in the development of cost-effective and efficient adsorbents. Studies demonstrate that zeolites, porous support-anchored metal oxides, and functionalized polymers exhibit promising phosphorus adsorption capacities [20,21,22]. However, these materials are often costly to synthesize and typically exist in powdered forms, limiting their applicability in fixed-bed reactors due to handling and separation challenges. Industrial wastes such as steel slag, fly ash, and sewage sludge have been explored for producing unburned ceramsite for phosphorus removal [23,24,25]. While these ceramsites show satisfactory performance, they often suffer from secondary pollution, poor stability, and low mechanical strength compared with non-sintered ceramsite. Although high-temperature sintering incurs certain additional costs, it provides multiple advantages: degradation of organic contaminants and pathogens, enhanced mechanical strength via crystalline restructuring, immobilization of hazardous metals, and formation of microporous structures that improve adsorption capacity [26]. Thus, sintered ceramsite derived from industrial waste presents a promising approach to address these challenges in phosphorus removal. Coal gasification slag (CGS), an industrial solid waste generated during the high-temperature (1200–1600 °C) coal gasification process, is primarily composed of mineral elements (e.g., Si, Al, Ca, Fe) and residual carbon [27]. In China, the coal chemical industry produces over 60 million tons of CGS annually, with less than 30% effectively utilized, resulting in significant land occupation and environmental risks, including air pollution from pungent gas emissions and water/soil contamination from runoff [28]. Nevertheless, the composition of CGS makes it an ideal candidate for ceramsite synthesis. Its carbon content promotes pore formation, while Ca, Al, and Fe components serve as active sites for phosphorus adsorption [29]. Therefore, utilizing CGS for ceramsite production not only offers a sustainable approach to waste management but also provides a cost-effective adsorbent for wastewater treatment. In this work, a ceramsite with alkali-supplying and calcium-releasing properties was prepared via high-temperature sintering, using CGS as the primary raw material. The ceramsite requires no additional chemicals during the phosphorus removal process and exhibits a self-adaptive function, especially in acidic wastewater. The research elucidates the synthesis process, characterizes the ceramsite’s physicochemical properties, and explores its phosphorus removal mechanism. By transforming CGS into a high-performance phosphorus removal material, this work addresses both the environmental pollution caused by industrial waste and the urgent demand for efficient phosphorus removal technologies. Furthermore, this research holds guiding and practical significance for the co-utilization of coal gasification slag and calcium-containing solid waste.

## 2. Experimental Section

### 2.1. Materials

The raw materials for synthesizing ceramsite are CGS, coal gangue, and calcium oxide. The chemical compositions of CGS and coal gangue are shown in Table 1. The CGS, obtained from a large coal chemical enterprise in Erdos, China, is a byproduct of the multi-nozzle opposed water coal slurry gasification process. Coal gangue was collected from Erdos in Inner Mongolia, China. With its high SiO_2_ and Al_2_O_3_ content, it serves as a binder and supplements the aluminum content in the mixture. The carbon present in both CGS and coal gangue contributes to pore formation in the ceramsite during the sintering process.

### 2.2. Ceramsite Preparation

The CGS and coal gangue were separately crushed, passed through a 140-mesh sieve, and stored for later use. The three raw materials were weighed and homogenized according to a specific mass ratio (component ratio shown in Table 2). A certain amount of the mixed sample was taken, and water was added at a ratio of 3.0–3.2 (ash-to-water ratio). The mixed sample was then pelletized in a pelletizing machine, with the pellet diameter controlled at 4–6 mm. The green pellets were dried in an oven at 110 °C for 8 h, followed by sintering in a high-temperature sintering furnace. The sintering temperature was set at 900–1160 °C with a heating rate of 10 °C/min and a sintering time of 60 min. After sintering, the pellets were cooled to room temperature naturally to obtain ceramsite. The ceramsite preparation process was optimized based on compressive strength, apparent porosity, and phosphorus removal capacity, which were the key performance indicators evaluated.

### 2.3. Phosphorus Removal from Simulated Wastewater

KH_2_PO_4_ was used to form simulated phosphorus-containing wastewater. Phosphate removal by ceramsite was studied in a series of batch experiments, dosed at 4 g/L. At a fixed interval, aliquots were taken from flasks, filtered using a 0.45 μm cellulose acetate filter, and analyzed for phosphate content. The phosphorus removal rate and phosphorus removal capacity were calculated by Equations (1) and (2), respectively.

*P_removal rate_* = (*C*_0_ − *C_t_*) * 100%/*C*_0_ (1)

*P_removal capacity_* = (*C*_0_ − *C_t_*) * *V*/*m* (2)

where C_0_ (mg·L^−1^) was the initial concentration of P, C_t_ (mg·L^−1^) was the concentration of P at time t, V (L) was the volume of simulated wastewater, and m (g) was the dose of ceramsite.

The effect of pH was investigated across a range of 3.0 to 10.0, with adjustments made using 0.1–1 mol/L HCl or NaOH. The kinetic experiments were conducted at a constant temperature of 20 °C and pH 7.0, with an initial total phosphorus (TP) concentration of 12.90 mg/L. The adsorption isotherms were determined at a constant temperature of 20 °C and pH 7.0, with TP concentrations ranging from 1.14 to 17.07 mg/L. Simulated wastewater with an initial TP concentration of 15 mg/L was selected to study the influence of coexisting anions on the phosphorus removal behavior of ceramsite. A 1.5 g portion of ceramsite was added to 250 mL of simulated wastewater containing specific concentrations of coexisting anions (HCO_3_^−^, NO_3_^−^, SO_4_^2−^, and Cl^−^), which were introduced by adding their respective sodium salts (NaHCO_3_, NaNO_3_, Na_2_SO_4_, and NaCl).The concentration of each coexisting ion mentioned above was set at 10, 20, 50, 100, 150, and 200 mg/L.

### 2.4. Hydrolysis Characteristics of Ceramsite

A batch experiment was conducted to investigate the release characteristics of calcium ions (Ca^2+^) and hydroxide ions (OH^−^) from ceramsites. A 1.5 g quantity of ceramsites was added to 250 mL of deionized water for reaction. The conical flask was placed in a constant-temperature (20 °C) water bath and shaken at a rotation speed of 150 r/min. At predetermined time intervals (20, 40, 60, 80, 100, 120, 180, 240, 300, 420, 540, 660, 780, 900 min), samples were collected to determine the concentration of calcium ions (Ca^2+^) and pH value of the deionized water.

### 2.5. Analytical Methods

The inorganic chemical composition of CGS and coal gangue were analyzed using an X-ray fluorescence spectrometer (XRF; PANalytical Ltd., Almelo, The Netherlands, Model MagiX PW2400). The carbon content of CGS and coal gangue were measured using an elemental analyzer (EA; PerkinElmer Ltd., Shelton, CT, USA, Model 2400II). Water absorption and apparent porosity of the ceramsites were determined according to ASTM methods. The compressive strength of the ceramsites was tested by an electronic universal tester (Shenzhen SANS Testing Machine Co., Ltd., Shenzhen, China, Model CMT5105). Solution pH was measured with a standard pH meter (Model PHS-3C, INESA, Shanghai, China). The Ca^2+^ concentration of the aqueous samples was determined by atomic absorption spectrophotometer (AAS, Persee TAS-990, Beijing, China). Phosphorus analysis was carried out with a UV–vis spectrophotometer using the molybdenum antimony method at 700 nm (Model UV-3600, Shimadzu, Kyoto, Japan). The phase composition of ceramsite was analyzed using an Advanced Powder X-ray Diffractometer with Cu-Kα radiation (XRD, Model D8 Advance, Bruker, Berlin, Germany). The surface morphology and elemental composition of ceramsite were analyzed using a scanning electron microscope and energy dispersive spectrometer (SEM, PI 89, Bruker, Germany; EDS, Elite TX, EDAX, Warrendale, PA, USA). Qualitative analysis of phosphate removal by ceramsite was carried out through X-ray photoelectron spectra (XPS, ESCALAB 250xi, Thermo, Waltham, MA, USA).

## 3. Results and Discussion

### 3.1. Compressive Strength, Water Absorption, and Apparent Porosity of Ceramsite

**Figure 1:**
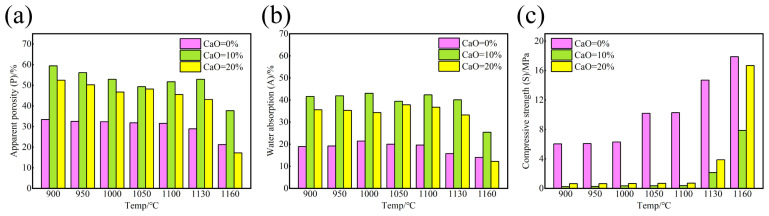
(**a**) Apparent porosity, (**b**) water absorption, and (**c**) compressive strength of ceramsite at different sintering temperature.

The apparent porosity, water absorption, and compressive strength of ceramsite at different sintering temperatures are shown in Figure 1a, Figure 1b, and Figure 1c, respectively. As the sintering temperature increased, the strength of ceramsite prepared with varying amounts of calcium oxide (0%, 10%, and 20%) rose, while its apparent porosity and water absorption decreased, a result of the sintering process. A significant increase in the strength of ceramsite was observed, particularly when the sintering temperature exceeded 1130 °C and calcium oxide was added. Overall, the variations in strength, porosity, and water absorption showed a strong correlation with the sintering temperature. Notably, compared to the ceramsite without calcium oxide addition, the strength of sintered ceramsite obtained with calcium oxide addition significantly decreased within the range of 900–1130 °C, while its porosity and water absorption significantly increased. Only at 1160 °C, the ceramsite obtained with 20% calcium oxide addition resulted in a dramatic increase in strength, accompanied by a sharp decrease in porosity and water absorption. These observations indicate that the addition of calcium oxide promoted the sintering and crystallization of the sample. The evolution of the crystalline phase also transformed the microstructure of the GCS from a dense to a porous structure [30,31]. The formation of substantial amounts of this porous structure significantly increased the porosity of the sintered ceramsite while reducing its strength [32]. However, at excessively high temperatures, the fluxing action of calcium oxide led to an increase in the liquid phase during sintering. This liquid phase filled part of the pores in the ceramsite, densifying it and thus substantially increasing its strength.

### 3.2. Phosphorus Removal Capacity of Ceramsite

**Figure 2:**
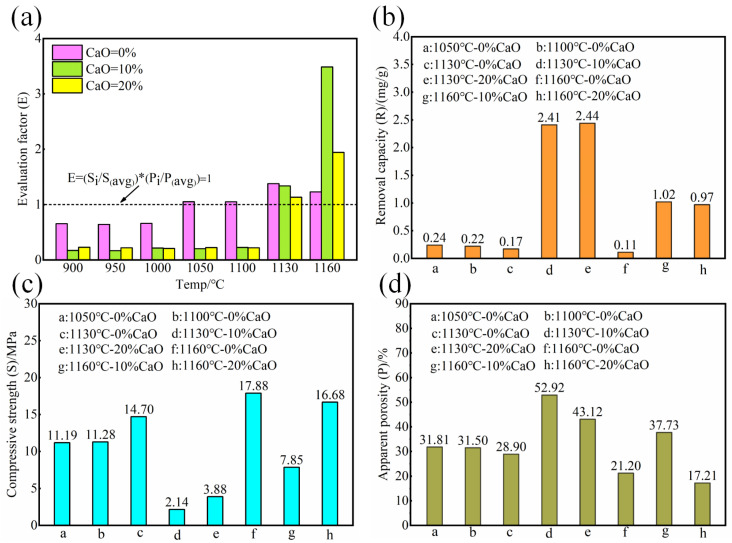
(**a**) The E values of the ceramsites at different sintering temperature. (**b**) The phosphorus removal capacity, (**c**) the compressive strength, and (**d**) the apparent porosity of ceramsite selected with an E value greater than 1.

Considering the actual application of water treatment media in fixed-bed systems, although high porosity in sintered ceramsite is beneficial for phosphorus removal, excessively low strength renders it impractical. Therefore, an evaluation factor was introduced to assess the performance of the synthesized ceramsite. The calculation of this evaluation factor is denoted as Equation (3). Figure 2a presents the E values of the ceramsites. Among all these ceramsites, those with E > 1 were selected for phosphorus removal tests. The results of the phosphorus removal tests and the corresponding strength and apparent porosity are shown in Figure 2b, Figure 2c and Figure 2d, respectively.

*E* = (*S_i_*/*S_(avg)_*) * (*P_i_*/*P_(avg)_*) (3)

where S_i_ (MPa) was the compressive strength of any individual ceramsite, S_(avg)_ (MPa) was the average strength of all ceramsites, P_i_ (%) was the apparent porosity of any individual ceramsite, and P_(avg)_ (%) was the average apparent porosity of all ceramsites.

The phosphorus removal experiment results revealed that, out of the eight optimized groups of ceramsite, those treated with 10% and 20% calcium oxide additions and sintered at 1130 °C and 1160 °C demonstrated superior phosphorus removal performance, coupled with higher porosity and reduced mechanical strength. In the absence of calcium oxide (CaO) addition, the optimized ceramsite maintained moderate porosity and high mechanical strength, but exhibited insufficient phosphorus removal capacity. Based on the aforesaid analysis, the formulation incorporating 20% CaO sintered at 1130 °C (designated CM-20-1130) achieved a balanced performance, demonstrating practical advantages in both mechanical strength and phosphorus removal capacity. The physicochemical properties of CM-20-1130 are systematically characterized in Table 3.

Notably, ceramsite synthesized with 20% calcium oxide addition and sintered at 1160 °C, despite its lower porosity (Figure 2d), demonstrated phosphorus removal capacity comparable to that of ceramsite produced with 10% calcium oxide addition at the same sintering temperature. This performance was fourfold higher than that of CaO-free ceramsite sintered at 1050 °C and 1100 °C. These results suggest that the phosphorus removal capacity of ceramsite is not solely dependent on porosity but is also critically linked to the mineral phases formed during CaO incorporation.

### 3.3. Crystalline Phase Analysis

**Figure 3:**
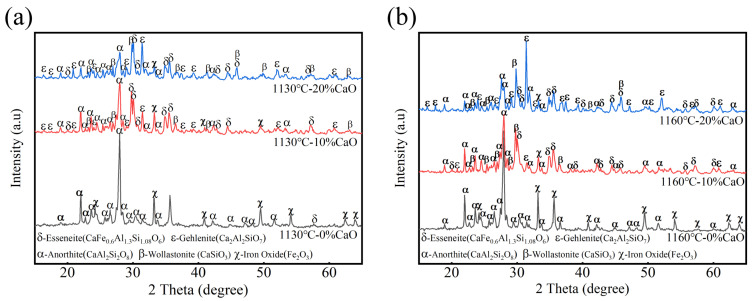
The XRD patterns of sintered ceramsite with different calcium oxide contents at (**a**) 1130 °C and (**b**) 1160 °C.

To further investigate the relationship between phosphorus removal capacity and the intrinsic properties of ceramsite, the mineral phase composition of selected sintered ceramsite samples was analyzed. The XRD characterization results are presented in Figure 3a and Figure 3b, respectively.

Without the addition of calcium oxide, at sintering temperatures of 1050 °C and 1100 °C, the main mineral phases of the ceramsite were anorthite (CaAl_2_Si_2_O_8_) and hematite (Fe_2_O_3_). The formation of a large amount of anorthite endowed the ceramsite with high strength. When calcium oxide was introduced, under sintering temperatures of 1130 °C and 1160 °C, the primary mineral phases of the ceramsite consisted of anorthite, gehlenite (Ca_2_Al_2_SiO_7_), wollastonite (CaSiO_3_), and esseneite (CaFe_0.6_Al_1.3_Si_1.08_O_6_) phases. At the corresponding sintering temperatures, the main mineral phases of the ceramsite without calcium oxide addition remained anorthite and hematite. Compared to a 10% addition, increasing the calcium oxide content to 20% significantly enhanced the formation of gehlenite and wollastonite. This effect was most pronounced at 1160 °C, where the abundance of these phases was substantially greater. By integrating analyses of ceramsite strength, porosity, and phosphorus removal capacity, the addition of calcium oxide within a certain temperature range promoted the formation of mineral phases such as gehlenite and wollastonite in the ceramsite. The development of these new phases may potentially enhance the phosphorus removal capability of the ceramsite. Research has shown that impurity elements such as sodium and potassium present during the sintering process can destabilize anorthite and gehlenite crystals. The same studies have directly demonstrated that gehlenite and wollastonite exhibit alkali-releasing and calcium-dissolving properties in aqueous solutions, with their capacities ranked in the following order: wollastonite > gehlenite > anorthite [33,34,35]. As a result, these mineral phases could undergo incongruent dissolution in aqueous solutions, releasing calcium ions (Ca^2+^) that exhibit affinity for phosphate ions (PO_4_^3−^), thereby enhancing the phosphorus removal performance of the ceramsite.

### 3.4. Hydrolysis Characteristics of the Prepared Ceramsite

**Figure 4:**
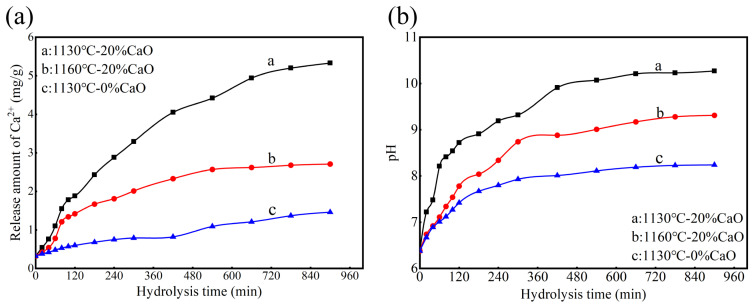
(**a**) The Ca^2+^ release in aqueous solutions and (**b**) the pH variations in aqueous solutions for the three tested ceramsite groups. Operating conditions: temperature, 20 °C; dose, 6.0 g/L; rotation speed, 150 r/min.

To further verify this characteristic of the prepared ceramsite, the Ca^2+^ release and pH variations in aqueous solutions over hydrolysis reaction time for the three tested ceramsite groups are presented in Figure 4a and Figure 4b, respectively. Ceramsite with 20% calcium oxide added, sintered at 1130 °C, released 5.40 mg/g of calcium ions and exhibited a pH of 10.31 in aqueous solution after 15 h of hydrolysis. In contrast, ceramsite without calcium oxide, sintered at the same temperature, released only 1.21 mg/g of calcium ions and showed a pH of 8.50 under identical hydrolysis conditions. The above results indicate that, compared with ceramsite with anorthite as the main crystalline phase, the ceramsite with gehlenite and wollastonite as the main crystalline phases obtained by sintering with the addition of calcium oxide has stronger hydrolysis capacity and correspondingly achieves stronger phosphorus removal capacity.

Moreover, the hydrolysis capacity of ceramsites is also related to their inherent physical properties. Ceramsites with a greater number of pores, looser structures, and lower compactness have a larger contact area with water and more pathways for ion diffusion, resulting in stronger hydrolysis capacity. Compared with the ceramsite sintered at 1130 °C with 20% calcium oxide added (with a porosity of 43.12%), although the ceramsite fired at 1160 °C produces a greater amount of anorthite and wollastonite, it has a lower porosity (17.17%), resulting in relatively weak hydrolysis capacity (with the calcium ion release amount and pH being 2.62 mg/g and 9.52, respectively), and correspondingly, it has relatively low phosphorus removal capacity. In summary, the phosphorus removal capacity of ceramsite is determined by its own hydrolysis capacity, which is related to the ceramsite’s phase composition and porosity. During sintering, the formation of gehlenite and wollastonite phases, supported by a highly porous structure, provides the ceramsite with a strong hydrolysis capacity. This enables the release of substantial calcium and hydroxide ions for phosphorus removal. As a result, in this study, part of the anorthite, gehlenite, and wollastonite could be dissolved incongruently in phosphate aqueous solution, as shown in Equations (4)–(6), releasing calcium ions and hydroxide ions to combine with phosphate, thereby achieving efficient phosphorus removal.

CaAl_2_Si_2_O_8_ + 3H_2_O = Ca^2+^ + 2OH^−^ + Al_2_[Si_2_O_5_][OH]_4_ (4)

(5) $$2{\mathrm{Ca}}_{2}{\mathrm{Al}}_{2}{\mathrm{SiO}}_{7}+9H_{2}O=4{\mathrm{Ca}}^{2+}+6{\mathrm{OH}}^{-}+2\mathrm{Al}[\mathrm{OH}{]}_{4}^{-}+{\mathrm{Al}}_{2}[{\mathrm{Si}}_{2}O_{5}]{\left[\mathrm{OH}\right]}_{4}$$

CaSiO_3_ + H_2_O = Ca^2+^ + 2OH^−^ + SiO_2_ (6)

### 3.5. Adsorption Kinetics and Isotherm

**Figure 5:**
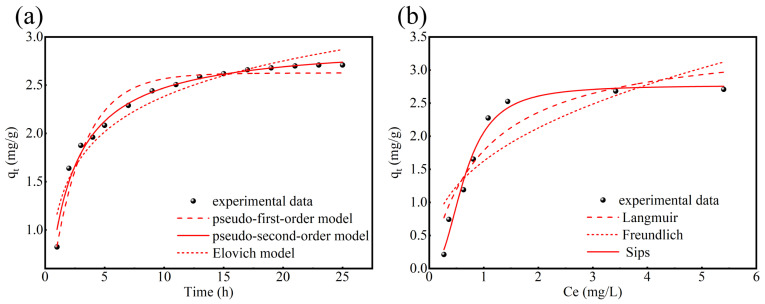
(**a**) Adsorption kinetics. Adsorption conditions: temperature, 20 °C; pH 7; dose, 4.0 g/L; initial concentration of TP, 12.90 mg/L. (**b**) Adsorption isotherm of phosphate on CM-1130-20. Adsorption conditions: temperature, 20 °C; pH 7; dose, 4.0 g/L; reaction time, 24 h.

P adsorption kinetics experiments were performed at adsorption times spanning 0–25 h (Figure 5a). The phosphate levels dropped from 12.90 mg/L to below 2.06 mg/L within the initial 20 h, and no further significant variation was noted over the following 5 h. In the initial stage (0–20 h), the increase in phosphorus removal capacity may be attributed to the sufficient active sites on the ceramsite. After 20 h, the active sites of the ceramsite were also saturated, resulting in no significant change in the phosphorus removal capacity of the ceramsite. To evaluate the phosphorus removal process, the experimental kinetic data were fitted to pseudo-first-order and pseudo-second-order models and an Elovich model, mathematically described by Equations (7), (8) and (9), respectively.

*q_t_* = *q_e_*(1 − *e^−k^*_1_*^t^*) (7)

(8) $$q_{t}=q_{e}^{2}k_{2}t\;/(1+q_{e}k_{2}t)$$

*q_t_* = *α* + *βlnt* (9)

where *t* (min) was the contact time; *q_e_* (mg·L^−1^) and *q_t_* (mg·L^−1^) were the p concentration at equilibrium and at time t; *k*_1_ (min^−1^) and *k*_2_ (mg^−1^ g^−1^·min^−1^) were the coefficient constant for the pseudo-first-order model and pseudo-second-order model, respectively; α (mg·g^−1^·min^−1^) was the initial adsorption rate for the Elovich model; and β (g·mg^−1^) was the Elovich model constant. Kinetic parameters for the phosphate adsorption to CM-20-1130 obtained using the three models are depicted in Table 4. Apparently, pseudo-second-order adsorption kinetics best describe the adsorption of P by CM-20-1130, indicating that the phosphorus removal is a chemisorption process involving electrons transferring between adsorbents and adsorbates [36].

The P adsorption isotherm was obtained by varying the initial concentration of TP from 1.14 to 17.07 mg/L (Figure 5b). The adsorption isotherm data were further analyzed using Langmuir and Freundlich and Sips models, which are mathematically represented by Equations (10), (11), and (12), respectively:

*q_e_* = *k_L_q_m_c_e_*/(1 + *k_L_q_m_c_e_*) (10)

*q_e_* = *k_F_q_m_c_e_*^1/*n*^ (11)

(12) $$q_{e}=k_{L}q_{m}c_{e}^{r}/{\left(1+k_{L}c_{e}\right)}^{\gamma }$$

where *q_m_* (mg·g^−1^) is the maximum adsorption capacity, *K_L_* (L·mg^−1^) is the Langmuir constant, *K_F_* (mg^1 − 1/*n*^·L^1/*n*^·g^−1^) and 1/*n* are the Freundlich constants, *γ* represents the heterogeneity of the adsorbent; the closer *γ* is to 1, the more uniform the surface of the adsorbent. The Langmuir isotherm assumes the presence of monolayer adsorption sites, while the Freundlich isotherm assumes multilayer adsorption to a heterogenous surface [37]. The Sips isotherm model is a combination of the Langmuir and Freundlich models. It is used to describe the adsorption process on non-uniform surfaces [38]. The Sips model fits the experimental data better than the Freundlich model and the Langmuir model (Table 4), suggesting that the adsorption process is relatively complex. The maximum phosphorus removal capacity of CM-20-1130 is 2.77 mg/g. Compared with several similar nitrogen and phosphorus removal materials reported in the literature (Table 5), the phosphorus removal capacity of CM-20-1130 is slightly lower. However, the ceramsite prepared in this study achieves a balance between phosphorus removal performance and essential engineering properties—exhibiting adequate mechanical strength (3.88 MPa), a critical attribute for engineering-scale applications. Notably, a comparable ceramsite reported in Reference [39] boasts a higher phosphorus removal capacity (7.79 mg/g) but substantially inferior mechanical strength (1.29 MPa).

### 3.6. Characteristics of CM-20-1130 Before and After Phosphorus Removal

**Figure 6:**
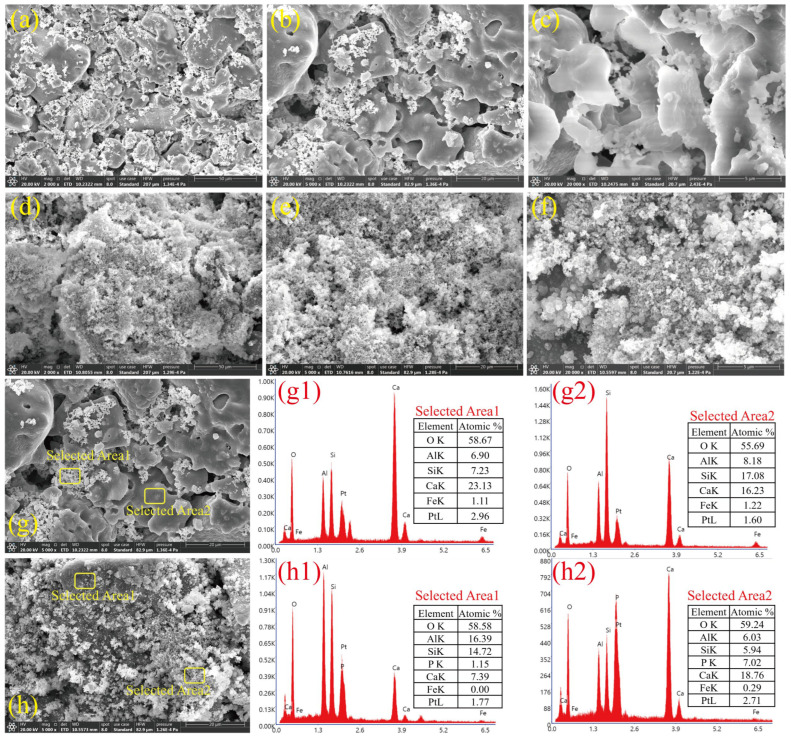
SEM images of CM-20-1130 (**a**–**c**) before and (**d**–**f**) after phosphorus removal. EDS mapping of CM-20-1130 (**g**) before and (**h**) after phosphorus removal. (**g1**): EDS analysis result of selected Area 1 on the CM-20-1130 before phosphorus removal, (**g2**): EDS analysis result of selected Area 2 on the CM-20-1130 before phosphorus removal; (**h1**): EDS analysis result of selected Area 1 on the CM-20-1130 after phosphorus removal, (**h2**): EDS analysis result of selected Area 2 on the CM-20-1130 after phosphorus removal.

In order to further clarify the phosphorus removal process of ceramsite, surface morphology and elemental characteristics of CM-20-1130 before and after phosphate removal were analyzed using SEM-EDS, and the results are depicted in Figure 6. The surface of the CM-20-1130 was rough, and many connected or open pores could be observed clearly. These pores were created by crystal growth and the release of accumulated gases, generated from the burning of carbon in coal gangue and coal gasification slag. After phosphate removal, a significant change was observed in the surface morphology of CM-20-1130. A large number of flocs were generated and attached to the surface of ceramsite, and the original pore structure was filled and covered by these flocs. The flocs attached to the surface of the CM-20-1130 after phosphorus removal may be precipitates formed by the interaction of phosphorus in the solution with Ca^2+^ and OH^−^ released from CM-20-1130. The pristine composite’s EDS mapping exhibited no characteristic phosphorus peak, while the phosphate-treated counterparts displayed a prominent P-specific signal. The phosphorus-specific peak, identified in the flocculent composites on CM-20-1130 after phosphorus removal (selected Area b-2), along with the corresponding EDS elemental mapping, confirmed the successful adsorption of phosphate onto the medium via precipitation.

**Figure 7:**
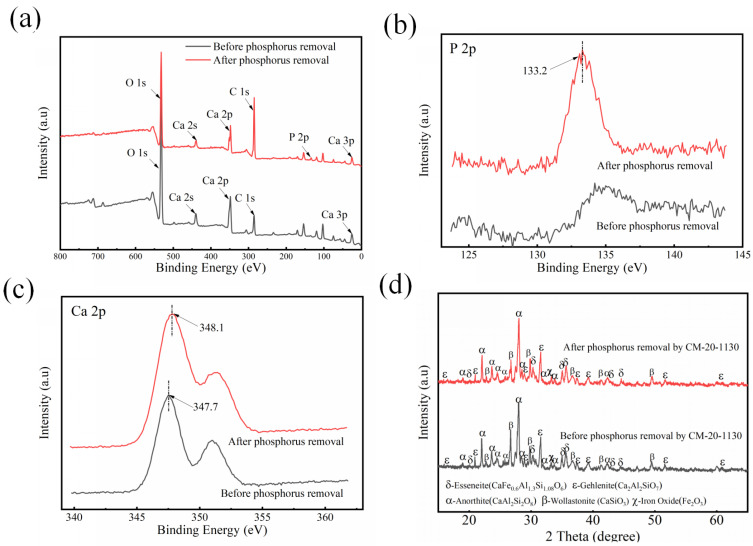
(**a**) XPS survey spectra, (**b**) P 2p high-resolution XPS spectra, and (**c**) Ca 2p high-resolution XPS spectra of CM-20-1130 before and after phosphorus removal. (**d**) XRD patterns of CM-20-1130 before and after phosphorus removal.

Qualitative analysis of phosphate removal by CM-20-1130 was also carried out through XPS and XRD. The XPS spectra of CM-20-1130 before and after P removal are depicted in Figure 7. The increased intensity of the P 2p XPS peak confirmed the adsorption of P by CM-20-1130. The P 2p spectra observed after P removal had a binding energy of 133.2 eV, which is about 0.7 eV lower than that of KH_2_PO_4_. The band at the binding energy of 133.2 eV in the P 2p spectrum should be attributed to the Ca–P complex [43]. The Ca 2p XPS peaks of CM-20-1130 were shifted about 0.4 eV toward higher binding energy (348.1 eV) after P removal, which also indicated that new chemical bonds were formed on the surface of CM-20-1130 [44]. The band at the binding energy of 347.4 eV in the P2p spectrum should be attributed to the calcium phosphate (Ca_3_(PO_4_)_2_) [45]. The hydrolytic properties of CM-20-1130 enabled the aqueous solution to maintain a stable pH of ~10.0 throughout the prolonged phosphorus removal treatment. According to the distribution diagram for phosphate as a function of pH, the alkaline matrix can promote the transformation of H_2_PO_4_^−^ to PO_4_^3−^ [23]. These species are more prone to combine with Ca^2+^ released from CM-20-1130, forming Ca_3_(PO_4_)_2_ precipitates. Furthermore, under highly alkaline conditions, Ca_3_(PO_4_)_2_ can undergo further transformation into hydroxyapatite (Ca_5_(PO_4_)_3_(OH)), a thermodynamically stable phase [46]. The relevant reactions are as follows:

3Ca^2+^ + 2H_2_PO_4_^−^ + 4OH^−^ = Ca_3_(PO_4_)_2_ ↓ + 4H_2_O (13)

5Ca^2+^ + 3H_2_PO_4_^−^ + 7OH^−^ = Ca_5_(PO_4_)_3_(OH) ↓ + 6H_2_O (14)

Based on the XRD (Figure 7d) and strength test (Table 6) results, the phase composition and mechanical strength of CM-20-1130 remained unchanged before and after phosphorus removal, with no phosphorus-containing peaks detected. A plausible reason is that the amount of precipitates formed on the ceramsite surface was insufficient for detection, as they were masked by the diffraction peaks of other phases. Second, the generated precipitates were amorphous and therefore not identifiable via XRD. From the perspective of material stability, the unaltered phase composition and retained mechanical strength of CM-20-1130 before and after phosphorus removal confirm its excellent structural stability.

**Figure 8:**
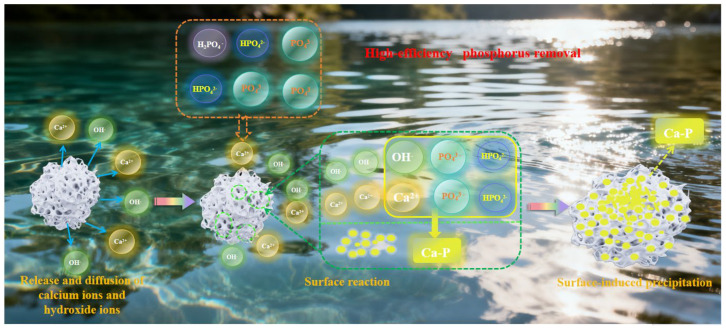
Phosphorus removal mechanism of CM-20-1130.

Although no phosphate-related characteristic peaks were detected via XRD, the plausible phosphorus removal mechanism (Figure 8) of CM-20-1130 can be inferred from the analysis of its properties before and after phosphorus removal. The phosphorus removal process of CM-20-1130 is dominated by adsorption/surface-induced coprecipitation. Specifically, phosphate ions in wastewater are first adsorbed onto the ceramsite surface through physical/chemical interactions, and then form Ca–P precipitates with calcium ions released from the ceramsite on its surface. With the continuous capture of phosphate ions, the precipitate layer gradually grows and thickens. Eventually, the precipitates form a heterogeneous coating on the ceramsite surface.

### 3.7. The Influence of pH and Coexisting Anions on Phosphorus Removal

**Figure 9:**
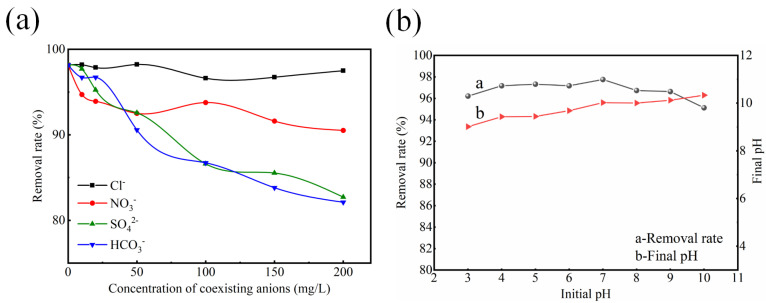
(**a**) Effect of coexisting anions (**a**) and pH (**b**) on phosphate removal by CM-20-1130. Operating conditions: reaction time, 24 h; temperature, 20 °C; pH, 7; initial TP concentration, 15 mg/L.

Anions such as NO_3_^−^, SO_4_^2−^, HCO_3_^−^, and Cl^−^ are commonly present in actual wastewater. These coexisting anions may compete with phosphate ions for the active adsorption sites of CM-20-1130, thereby reducing the phosphorus removal rate. The effect of coexisting anions on the phosphorus removal rate of CM-20-1130 is shown in Figure 9a. The phosphorus removal rate of CM-20-1130 is weakly affected by Cl^−^ and NO_3_^−^. Under different ion concentration conditions, the phosphate removal rate remains above 93%, which indicates that NO_3_^−^ and Cl^−^ do not compete with PO_4_^2−^ in the process of phosphate adsorption by CM-20-1130.The presence of SO_4_^2−^ and HCO_3_^−^ has a slight impact on the phosphorus removal rate, especially under high concentration conditions. This phenomenon may be related to the mechanism of surface precipitation. However, the solubility product constant of calcium phosphate is much lower than that of calcium sulfate and calcium carbonate. Even under the conditions of high concentrations of sulfate ions and bicarbonate ions, the phosphate removal efficiency still remains above 82%, which reflects the good selectivity of CM-20-1130 for phosphate removal.

The effect of pH on the rate of phosphorus removal by CM-20-1130 is shown in Figure 9b. When the initial pH of the simulated wastewater ranges from 3 to 10, the phosphorus removal efficiency of the ceramsite remains at a high level (>95%), and the pH of the simulated wastewater after phosphorus removal is maintained between 9.0 and 10.3. This can be attributed to the dissolution of wollastonite and gehlenite, which are unstable in acidic environments, leading to the release of Ca^2+^ ions that facilitated phosphorus removal [47]. In addition, most of the OH^−^ released from the ceramsite was reacted with H^+^ to increase the pH of the aqueous solution. These findings demonstrate that ceramsite possesses an intrinsic pH buffering capacity, exhibiting remarkable resistance to acid–base fluctuations in phosphorus removal performance.

## 4. Conclusions

In the process of synthesizing sintered ceramsite from coal gasification slag, the addition of calcium oxide promotes the sintering and crystallization of ceramsite at 900–1130 °C. This process makes the ceramsite form a large number of pore structures, leading to an increase in the apparent porosity of the ceramsite while causing a decrease in its compressive strength.

During the sintering process, increasing calcium oxide content and sintering temperature drives the transformation of the ceramsite’s main crystalline phases from anorthite and hematite to gehlenite, anorthite, wollastonite, and esseneite. Under optimal conditions (1130 °C, 20% CaO addition, 1 h sintering), the synthesized ceramsite exhibits an apparent porosity of 43.12%, compressive strength of 3.88 MPa, apparent density of 1.084 g/cm^3^, and water absorption rate of 33.20%.

The phosphorus removal capacity of ceramsite is determined by its hydrolysis characteristics, with a maximum phosphorus removal capacity of 2.77 mg/g. The formation of a large amount of gehlenite and wollastonite phases as well as the high porosity endow the ceramsite with certain hydrolysis ability. The calcium ions and hydroxide ions released through hydrolysis in aqueous solution enable the ceramsite to have a certain pH self-regulation function, allowing it to be used within a relatively wide pH range and exhibiting good selectivity for phosphate.

The phosphorus removal process of ceramsite kinetically aligns with the pseudo-second-order model, while its adsorption isotherm conforms to the Sip model. In simulated wastewater containing phosphorus, calcium ions (Ca^2+^) and hydroxide ions (OH^−^) released from the gehlenite and wollastonite phases in ceramsite react with phosphate ions (PO_4_^3−^) to form calcium-phosphate precipitates on the ceramsite surface, enabling efficient phosphorus removal.

## Figures and Tables

**Table 1:** Chemical composition of the raw materials (wt.%).

Raw Material	SiO_2_	Al_2_O_3_	Fe_2_O_3_	CaO	MgO	SO_3_	TiO_2_	K_2_O	Na_2_O	C/%
CGS	46.90	16.78	14.59	15.44	1.25	0.89	0.77	1.66	1.4	11.71
coal gangue	44.64	51.66	0.74	0.25	0.21	0.96	0.86	0.20	0.32	3.23

**Table 2:** Composition ratio of ceramsite.

GCS/%	Coal Gangue/%	CaO/%
75	25	0
65	25	10
55	25	20

**Table 3:** Physical properties of CM-20-1130.

Compressive Strength (MPa)	Apparent Porosity (%)	24 h Water Absorption (%)	Apparent Density (g/cm^3^)
3.88	43.12	33.20	1.084

**Table 4:** Kinetic and Isotherm model fitting parameters for the adsorption of phosphate by CM-20-1130 (298 K).

Adsorption Kinetics			Adsorption Isotherm		
Pseudo-first-order	*q_e,cal_* (mg/g)	2.624	Langmuir	*q_m_* (mg/g)	3.495
	*K*_1_ (min^−1^)	0.3805		*k_L_* (L/mg)	1.040
	R_1_	0.9566		*R*_1_	0.848
Pseudo-second-order	*q_e,cal_* (mg/g)	2.9522	Freundlich	1/*n*	0.387
	*K*_2_ (min^−1^)	0.1744		*K_F_* (mg^1−1/*n*^·L^1/*n*^·g^−1^)	1.624
	*R*_2_	0.9827		*R*_2_	0.683
Elovich	*α* (mg·g^−1^·min^−1^)	1.1613	Sips	*q_m_* (mg/g)	2.770
	*β* (g·mg^−1^)	0.5301		*k_L_* (L/mg)	2.888
	*R_3_*	0.9372		*γ*	2.462
				*R_3_*	0.976

**Table 5:** Comparison of capacities of different phosphorus-removing materials.

Phosphorus Removal Materials	Raw Materials	Q_max_ (mg/g)	References
Ceramsites	Fly ash, molybdenum tailings	7.79	[39]
Ceramsites	Solid waste	0.42	[19]
Ceramsites	Solid waste	2.49	[40]
Clay/Biochar composite	Reed straw, clay	0.75	[41]
Zeolite ceramsite	Fly ash	8.9	[42]
Granular ceramic	Loess	1.27	[21]
Ceramsites	CGS, coal gangue	2.77	This study

**Table 6:** Compressive strength of CM-20-1130 before and after phosphorus removal.

CM-20-1130	Compressive Strength (MPa)	Apparent Porosity (%)
Before Phosphorus Removal	3.85	43.09
After Phosphorus Removal	3.86	39.12

## Data Availability

Data will be made available upon request.
